# Interesting Case of Vomiting

**Published:** 1879-05-20

**Authors:** Da Costa


### *INTERESTING CASE OF VOMITING.*

A CLINICAL LECTURE FROM PROF. DA COSTA.

I want to say a few words to you regarding vomiting as a symptom in gastric disease.

Æt. of patient twenty-five. Family history not good. Sister and father both died of phthisis. She herself was always healthy. Began to menstruate at age of seventeen and stopped menstruating at twenty. Since then the menses have been very irregular. She married at the age of eighteen and was a widow at twenty-one. You will therefore notice that the irregularity of menstruation has existed not only during marriage, but also before and since.

She comes into the hospital for the treatment of what is apparently a very serious difficulty, viz: she has been vomiting constantly for nearly a year. She has been in the hospital for only a week. She has been vomiting incessantly; has never retained more than one meal during the course of the day for the past year. The vomiting always begins the instant after her meal is over. She does not have much vomiting or nausea between her meals.

A year ago she was stout and healthy, but the vomiting has rendered her thin and pale. Though not so this morning, it is fair to state that she has picked up wonderfully within the last few days, and you will, no doubt, want to know what has been done.

In the first place, I had the vomited matter examined, with but negative results. There were no sarcinæ, nothing but mucus mixed with the contents of the stomach. Occasionally the patient has been disturbed by vomiting mucus in her sleep.

Together with the vomiting you notice that she has a slight, irritative cough. This cough has troubled her ever since the vomiting began. Joined with the cough there is no expectoration.

Before going any farther, however, I will examine the gastric and intestinal organs. Her tongue is slightly coated and flabby, and there is some tenderness in the epigastric region and along the spine, particularly at about the middle point of the spine. There is, however, no appearance of a tumor. The soreness in the epigastrium is general, and is not localized in particular spots. The bowels are constipated; the respiratory sounds are normal. There is no albumen or abnormal ingredients in the urine. There is no fever, and the temperature is normal. The urine is acid and of the usual color, with a specific gravity of 1025.

What has been the cause of the vomiting? What remedy is it which has stopped the vomiting in three days?

When I first saw the woman in the wards and heard of the incessant vomiting, I first thought it was a case of irritable stomach in a young woman, connected with gastric ulcer. The epigastric soreness, the age of the patient, the appearance of the tongue, the disordered menstruation, the sore point in the spine, all tended in that direction.

I soon gave up the idea, however. Gastric ulcer gives rise to local soreness; here the soreness was general. Gastric ulcer is attended by hemorrhage and pain upon taking food, which was not the case here. The two most prominent symptoms of ulceration were absent. I rejected the idea.

Then there came up a point of experience in my mind—one case similar had happened not long since in my own practice, several I had seen in consultation. In one case the vomiting had reduced the patient almost to the verge of the grave; nothing stayed on her stomach. In her case, the irritation was reflected upon the stomach from a uterine malady—a slight flexion. It was not very different from the sympathetic vomiting of pregnancy. Moreover, there was in that case a certain amount of gastric disease—a catarrhal affection of the stomach came on as a complication of the nervous affection. In the light of that experience I began to suspect the same condition here. As a result of examination we found retroflexion of the uterus. The whole case was cleared up at once. It was reflex vomiting with a certain amount of gastric catarrh, lasting for a year, although the woman had taken the greatest care with her diet, etc.

When she was first admitted she was put upon lime-water and milk, but there was no effect produced upon the vomiting. How did we check it? It was not by diet alone.

Again experience came to my assistance, and I determined to try the application of ice to the spine as a systematic treatment, every few hours. The ice was applied and left on until it chilled the patient and made her skin cold. The application was often repeated—as often as she could bear it. Its effects were admirable. No other treatment was necessary. The vomiting stopped almost at once.

You will not always be so successful with this remedy alone. Is there nothing which we can combine with the ice? Must we depend upon it alone? By no means. Bromide of sodium in doses of ten or fifteen grains thrice daily is very effectual. It lessens the reflex irritation, and is not rejected by the stomach. An occasional purge by an enema, or some bitter-water, is often desirable. Subsequently, if the case lingers, use blisters to the spine. Do all this irrespective of the local uterine treatment, for the reduction of the uterine displacement will not always stop the vomiting at once—the cause is removed, but not the habit.

To-day I shall introduce a pessary. Already the girl's diet has been increased, and she is beginning to retain solid food.

What else can we use in such cases to soothe the stomach? Pepsin is very valuable as soon as the vomiting has been stopped. The dose of saccharated pepsin is five grains thrice daily. The diet all this while should be gradually increased; only small quantities of food being given, but these frequently.

This condition is very similar to hysterical vomiting. There is no manifestation of hysteria here. The two states are parallel, but not identical. In an hysterical case, the results of treatment would not be so good.—*N. Y. Med. Reporter.*

